# Impact of polymorphisms in microRNA biogenesis genes on colon cancer risk and microRNA expression levels: a population-based, case-control study

**DOI:** 10.1186/s12920-016-0181-x

**Published:** 2016-04-23

**Authors:** Lila E. Mullany, Jennifer S. Herrick, Roger K. Wolff, Matthew F. Buas, Martha L. Slattery

**Affiliations:** Department of Internal Medicine, University of Utah, School of Medicine, Salt Lake City, UT USA; Division of Public Health Sciences, Fred Hutchinson Cancer Research Center, Seattle, WA USA

**Keywords:** miRNA, Biogenesis, SNP, Colon, Cancer, Risk

## Abstract

**Background:**

MicroRNAs (miRNAs) have been implicated in the incidence and progression of cancer. It has been proposed that single nucleotide polymorphisms (SNPs) influence cancer risk due to their position within genes involved in miRNA synthesis and regulation.

**Methods:**

Genes directly and indirectly involved in miRNA biogenesis were identified from the literature. We then identified SNPs within these regions. Using genome-wide association study data we evaluated associations between biogenesis-related SNPs with colon cancer risk and their corresponding mRNA expression in normal colonic mucosa and carcinoma and difference in expression between the two tissues. SNPs that were associated with either altered colon cancer risk or with mRNA expression were evaluated for associations with altered miRNA expression.

**Results:**

Eleven SNPs were associated (*P* < 0.05) with colon cancer risk, and two of these variants remained significant after correction for multiple comparisons (P_Holm_ < 0.05): rs1967327 (*PRKRA*) (OR_dom_ = 0.78, 95 % CI 0.66–0.92) and rs4548444 (*MAPKAP2*) (OR_rec_ = 1.67, 95 % CI 1.12–2.48). Of these two SNPs, rs4548444 (*MAPKAP2*), was associated with significantly altered miRNA expression levels in normal colonic mucosa, with nine miRNAs upregulated among individuals homozygous rare (GG) for rs4548444. One SNP associated with cancer prior to adjustment for multiple comparisons, rs11089328 (*DGCR8*), was associated with altered levels of hsa-miR-645 in differential tissue under the dominant model. Three SNPs, rs2740349 (*GEMIN4*) in carcinoma tissue, and rs235768 (*BMP2*) and rs2059691 (*PRKRA*) in normal mucosa, were significantly associated with altered mRNA expression levels across genotypes after multiple comparison adjustment. Rs2740349 (*GEMIN4*) and rs235768 (*BMP2*) were significantly associated with the upregulation of six and nine individual miRNAs in normal colonic mucosa, respectively.

**Conclusion:**

Our data suggest that few of the SNPs in biogenesis genes we evaluated alter levels of mRNA transcription or colon cancer risk. As only one SNP both alters colon cancer risk and miRNA expression it is likely that SNPs influencing cancer do not do so through miRNAs. Because the significant SNPs were associated with downregulated mRNAs and upregulated miRNAs, and because each SNP was associated with unique miRNAs, it is possible that other mechanisms influence mature miRNA levels.

## Background

Mature microRNAs (miRNAs) are non-coding RNA molecules, ~22 nucleotides (nt) in length [1–5], which act as endogenous, post-transcriptional regulators of messenger RNAs (mRNAs). By binding to complementary mRNA molecules, they are able to impede mRNA translation or cause mRNA degradation [6], depending on the degree of complementarity shared between the miRNA and mRNA [7]. As such, miRNAs alter the translated protein product levels of these genes within the specific biological tissues and diseases [8] in which they are expressed. It has been suggested that single nucleotide polymorphisms (SNPs) within miRNA gene regions that are associated with cancer risk may function by altering miRNA expression [9]. Similarly, SNPs within any of the genes regulating miRNA biogenesis would have the potential to alter the expression of mature miRNAs and subsequently cancer risk [10].

There are many transcriptional and post-transcriptional [11] modification steps necessary for producing mature miRNAs. Genes directly involved in splicing, exporting and RNA editing events [12], as well as accessory proteins [13], have the potential to impact miRNA expression levels. MiRNA biogenesis can be broken down into four main categories of events: transcription, nuclear processing, cytoplasmic processing and RNA-induced silencing complex (RISC) formation and loading [11, 12]. We provide a brief overview of miRNA biogenesis.

### Transcription

Primary-miRNAs (pri-miRNAs) are transcribed by RNA polymerase II [11, 14], and produce transcripts of up to 1 kilobase (kb) in length [11]. They consist of potentially more than one co-transcribed precursor miRNA sequence (pre-miRNA), as illustrated by miRNAs organized in a polycistronic cluster [15]. Transcription factors, such as *TP53* and *MYC*, and epigenetic regulation, such as histone modifications, can alter miRNA transcription [11]. Once transcribed, pri-miRNAs fold into hairpin structures, consisting of a stem sequence (~35 nt), a terminal loop and single-RNA strands at both the 5′ and 3′ ends [11, 12]. These hairpin structures are recognized as substrates for the ribonuclease (RNase) III enzymes Drosha and Dicer [13].

### Nuclear processing

Pre-miRNAs are generated by the cleavage of the pri-miRNA in the nucleus of the cell by the Microprocessor, which is comprised of Drosha and DiGeorge Syndrome Critical Region 8 (DGCR8) [12, 16]. The stem and tail regions of the pri-miRNA are important for the first cleavage: DGCR8 binds to the pri-miRNA using these regions and serves to align Drosha with the pri-miRNA to cleave at the appropriate site, which is approximately 11 base pairs (bps), or 1 helical turn [17], away from the junction of the single and double strands within the hairpin [12]. GSK3β has been shown to phosphorylate Drosha in the cytoplasm before it enters the nucleus, which enables it to be localized to the nucleus and contribute to miRNA biogenesis [18]. Drosha-mediated cleavage results in a hairpin of ~65–70 bps [11, 13] and leaves behind a ~2 nt overhang on the 3′ end, which is characteristic of RNase III enzymes [19], and makes it possible for the pri-miRNA to be recognized by and subsequently transported out of the nucleus and into the cytoplasm by the Exportin-5 (XPO5) [17, 19] -Ran-GTP complex [7, 12]. Adenosine-Deaminase, RNA-Specific (ADAR) genes act on pre-mRNA transcripts, editing adenosine to inosine, which acts similarly to guanosine during translation, potentially altering protein function [20]. Because ADARs work on nuclear, double-stranded RNA structures, it is likely that they edit pri-miRNA transcripts prior to their export from the nucleus. Also acting in the nucleus, transforming growth factor-β (TGF-β) and bone morphogenetic protein factors (BMPs) have been shown to increase Microprocessor activity through the recruitment of SMAD proteins and DDX5 to the miRNA transcript, enhancing processing by Drosha [12] and potentially increasing miRNA expression.

### Cytoplasmic processing

Once in the cytoplasm, the pre-miRNA is cleaved by Dicer, which forms a complex with TRBP (or TARBP2, trans-activating response RNA binding protein) complex to produce the two strands of miRNA duplex [12]. Dicer removes the loop of the pre-miRNA hairpin, cleaving about 2 helical turns from the base of the hairpin [17], leaving behind ~22 bps of the miRNA/*miRNA duplex (where * is pronounced “star” [17]) [12]. Serine phosphorylation of TRBP by mitogen-activated protein kinase (MAPK)/extracellular regulated kinase (ERK) has been shown to stabilize TRBP [13]. Dicer activity has been shown to be impaired by decreased levels of TRBP [13]; as such SNPs within TRBP or MAPK/ERK could potentially lead to altered processing of pre-miRNAs.

### MiRISC formation and loading

Of the two strands of the mature miRNA duplex generated by Dicer cleavage, the guide strand is typically the strand with the more unstable 5′ end [12, 21]. This strand is selected by the Argonaute (AGO1-4) protein and loaded into the RISC with the help of the RISC-loading complex, which has been proposed to be comprised of Dicer and TRBP [11, 13]. The other strand, the star strand, is usually degraded [17]. Loading of the guide strand into the complex stabilizes the miRNA [22], protecting it from degrading nucleases within the cytoplasm, and enables it to bind to its target mRNA to induce silencing of the transcript. Helicases, such as the DEAD-Box proteins (GEMIN3 and p68), GEMIN4 and MOV10, are important for miRNA duplex unwinding and miRISC formation and activity [12].

There are additional accessory genes associated with miRNA biogenesis. Trinucleotide repeat-containing proteins (TRNC6A or GW182, TRNC6B, TRNC6C) and fragile X mental retardation protein (FMRP, encoded by *FMR1*) are correlated with the presence of cytoplasmic p-bodies, which are involved in mRNA degradation by miRNAs, and can impact the effect of miRNAs on their targets [13]. MiRNA stability, and therefore expression, has been linked to mRNA target presence and ability to be bound to the miRISC [22, 23]; as such, SNPs within genes that regulate mRNA degradation may impact mature miRNA expression levels. LIN28B regulates Microprocessor activity and LIN28 regulates dicing of pre-let-7 through the regulatory sequence “GGAG” in the pre-miRNA’s terminal loop [12, 13, 24, 25]; LIN28 binds to this region and recruits terminal uridine transferase (TUT4 or ZCCHC11), which subsequently uridylates the pre-miRNA and inhibits processing by Dicer [13, 24, 25].

In this study, we investigate the risk of developing colon cancer associated with SNPs found within miRNA-biogenesis genes. We also analyze each SNP with mRNA expression of its corresponding gene in normal colonic mucosa, carcinoma tissue and difference in expression between normal colonic mucosa and carcinoma to determine if these SNPs alter mRNA transcription. Based on these results, we analyze those SNPs associated with either altered colorectal cancer (CRC) risk or with altered mRNA expression of its corresponding gene with miRNA expression in normal colonic mucosa and differential miRNA expression between carcinoma tissue and normal colonic mucosa across genotypes. As genes involved in the biogenesis of miRNAs are involved in production of virtually all miRNAs, we hypothesize that SNPs in these genes will alter mRNA expression by causing aberrant transcription as well as alter colon cancer risk through altered levels of miRNA expression.

## Methods

### Study population

The study population consisted of individuals previously enrolled in a study of Diet, Lifestyle and Colon cancer at the University of Utah and the Kaiser Permanente Medical Research Program (KPMRP) [26] for whom Genome Wide Association Study (GWAS) and miRNA expression data were available. Study subjects included incident cases of colon cancer between the ages of 30 and 79 who were non-Hispanic white, Hispanic or African American and were able to provide a signed informed consent prior to participation in the study. All stages of tumor were included in the study population and in this analysis. The study was approved by the University of Utah Institutional Review Board for Human Subjects.

### miRNA processing

RNA (miRNA) was extracted from formalin-fixed paraffin embedded tissues and processed as previously described [27]. 100 nanograms (ng) total RNA was labeled with Cy3 and hybridized to Agilent Human miRNA Microarray V19.0 and were scanned on an Agilent SureScan microarray scanner model G2600D using Agilent Feature Extract software v.11.5.1.1. Data were required to pass stringent quality control (QC) parameters established by Agilent that included tests for excessive background fluorescence, excessive variation among probe sequence replicates on the array, and measures of the total gene signal on the array to assess low signal. If samples failed to meet QC standards, the sample was repeated. If a sample failed QC assessment a second time the sample was deemed to be of poor quality and was excluded from down-stream analysis. The Agilent platform was found to be highly reliable (*r* = 0.98) and had reasonable agreement with NanoString [28] and excellent agreement with quantitative reverse transcription polymerase chain reaction (qRT-PCR) [29]. If data were missing from normal colonic mucosa but the tumor tissue was successfully scanned (*N* = 60), we imputed values for normal mucosa as previously described in [30]; this method of imputation has yielded results with high accuracy. To minimize differences that could be attributed to the array, amount of RNA, location on array or other factors that could erroneously influence expression, total gene signal was normalized by multiplying each sample by a scaling factor which was the median of the 75th percentiles of all the samples divided by the 75th percentile of each individual sample [31]. This scaling factor was implemented using SAS 9.4.

### RNA-Seq sequencing library preparation

Total RNA was available from 197 carcinoma and normal mucosa pairs. These samples were taken from the study subjects used for miRNA analysis and were extracted, isolated and purified in the same manner as previously described [27]. RNA library construction was done with the Illumina TruSeq Stranded Total RNA Sample Preparation Kit with Ribo-Zero. The samples were then fragmented and primed for complementary DNA (cDNA) synthesis, adapters were then ligated onto the cDNA, and the resulting samples were then amplified using PCR; the amplified library was then purified using Agencount AMPure XP beads. A more detailed description of the methods can be found in our previous work [32]. Of these, 175 passed QC based on acceptable number of sequence reads for both carcinoma tissue and normal mucosa. Of these, 71 subjects also had GWAS data available for comparison with mRNA and miRNA expression data in carcinoma tissue, 67 for normal colonic mucosa, and 61 for the difference between normal colonic mucosa and carcinoma tissue.

### RNA sequencing and data processing

Sequencing was done using an Illumina TruSeq v3 single read flow cell and a 50 cycle single-read sequence run was performed on an Illumina HiSeq instrument. Reads were then aligned to a sequence database containing the human genome (build GRCh37/hg19, February 2009 from genome.ucsc.edu) and alignment was performed using novoalign v2.08.01. Python and a pysam library were used to calculate counts for each exon and UTR of the genes using a list of gene coordinates obtained from http://genome.ucsc.edu. We dropped features that were not expressed in our data or for which the expression was missing for the majority of samples. A more detailed description of the methods can be found in our previous work [32].

### Targeted SNPs from GWAS for biogenesis genes

#### SNP identification and bioinformatics analysis

A literature search was conducted to identify genes involved in miRNA biogenesis, as well as SNPs within biogenesis genes that alter cancer risk, by searching PubMed and standard search engines for peer-reviewed articles containing keywords “miRNA” and “biogenesis”, and the subsequent addition of other words that appeared in preliminary searches or those that would yield more specific results such as “degradation”, “processing”, “polymorphism”, “SNP” and “cancer” [10–13, 16, 17, 20, 22, 23, 33, 34]. This included the all genes directly associated with miRNA biogenesis (such as *DROSHA*, *DICER*, *DGCR8*, *TARBP2*, *AGO1-4, GEMIN3/4*) as well as accessory genes, or genes indirectly associated with biogenesis (such as *ADARs*, *BMPs*, *SMADs*, *FMR1, DDXs, DHXs, LIN28, MOV10, ZCCHC11*, *MAPKs, GSK3β, p38, p54*). NCBI SNP (http://www.ncbi.nlm.nih.gov/snp/) [35] was used to identify SNPs within these genes. We filtered results for ‘only Human active’, ‘snp’, with a custom minor allele frequency (MAF) range of 0.01–1.0; some gene-SNP associations were identified from the literature and as such were not screened for these criteria. This list represents the majority of SNPs within major human miRNA biogenesis genes. To better evaluate the impact of these SNPs on the expression of the proteins, we utilized Ensembl’s Variant Effect Predictor (VEP) tool (http://grch37.ensembl.org/info/docs/tools/vep/index.html) [36]; we used the archived, GRCh37, which corresponds to our GWAS and mRNA data.

#### GWAS genotyping

GWAS data were obtained using Illumina HumanHap 550, 610 K as part of the GECCO study and has been described previously [37]. Imputation to HapMap2 Release 24 was performed using MACH, which was imputed to HapMap Release 22 using BEAGLE. All SNP coordinates were defined using hg19/GRCh37. We excluded SNPs from consideration that failed Illumina quality measures or standard quality control procedures [38]. We further excluded SNPs that showed limited variability in our data. In total, we evaluated 219 SNPs with colon cancer risk; those that were significant were then evaluated with all miRNAs expressed in normal colonic mucosa in at least 5 % of the population. The final list of included SNPs can be seen in Table 1.

**Table 1:** Included SNPs and action and location of involved miRNA biogenesis genes

Gene^b^	SNP ID^a^	Active Loc.^d^	Process
*ADAR*	rs11264222, rs1127309, rs1127313, rs1127314, rs1127317, rs2131902, rs2229857, rs2335230, rs3766925, rs376692, rs4845384	N	Pre-miRNA Editing
*AGO1*	rs636832, rs11584005^f^, rs595055^c^	C	miRISC Formation/Loading
*AGO2*	rs10087629, rs1060832, rs11166984, rs11166985, rs13276958, rs1878478, rs2176397, rs2271735, rs2292775, rs2292780, rs2293939, rs2944761, rs2944767, rs2944776, rs2977469, rs2977477, rs2977490, rs3735805, rs3864659, rs3928672, rs4961278, rs6985156, rs7009635, rs7824304, rs7843258, rs7460^a^, rs2944760		
*AGO3*	rs10796876, rs274732, rs4351606		
*AGO4*	rs12044203, rs12119583		
*BMP2*	rs15705, rs2273073, rs235768, rs3178250	N	Recruitment of SMAD/Enhancement of Drosha
*BMP3*	rs3733549		
*BMP4*	rs17563, rs76335800^f^		
*BMP6*	rs1043784, rs1044104, rs7764128, rs9505298		
*DCP1B*	rs2240610, rs6489338	C	Localization with AGO & P-bodies/mRNA Decapping for Degradation
*DDX10*	rs10890898, rs2726920	C	Pre-miRNA Unwinding/Possible Drosha Processing Enhancement. DDX5-DDX17 Required for Recognition of pri-miRNA By Drosha.
*DDX11*	rs1808348, rs2075321, rs7953706, rs9750		
*DDX13 (SKIV2L)*	rs437179 (rs438999, rs449643 only SKIV2L)		
*DDX20*	rs11584657, rs197381^a^, rs197392, rs197412, rs197414, rs538779		
*DDX5 (p68)*	rs1991401, rs1140409		
*DGCR8*	rs11089328, rs1558496, rs1640297, rs17817767, rs2073778, rs2286926, rs2286928, rs41281429, rs417309, rs9606248	N	Pri-miRNA Cleavage
*DHX15*	rs6841898	C	May Interact with AGO1/2, Dicer, TRBP/Facilitate mi/siRNA Loading (DHX6,9).
*DHX16*	rs3130000		
*DICER1*	rs10149095, rs1057035, rs11160231, rs11622643, rs11624081, rs1209904, rs12323635, rs12897280, rs13078, rs17091855, rs3742330, rs4513027, rs1110386, rs11621737	C	Pre-miRNA Cleavage
*DROSHA*	rs10052174, rs10067066, rs10068052, rs10719, rs11748548, rs13169883, rs13183642, rs16901109, rs16901165, rs17408227, rs17408716, rs17409624, rs17409803, rs17410035, rs17485323, rs2279797, rs2287584, rs3792830, rs3805525, rs4867329^f^, rs4867339, rs493760, rs524138, rs573010, rs639174, rs6450848, rs673019, rs6878195, rs6884823, rs7447423, rs7712155, rs7712436, rs7719666, rs13175906^a^, rs2330696^a^, rs6883386^a^, rs7702984^f^	N	Pri-miRNA Cleavage
*FMR1*	rs25704^f^	C	P-bodies and mRNA Degradation
*GEMIN4*	rs1045481, rs1062923, rs2251689, rs2291778, rs2740348, rs2740349, rs3744741, rs7813, rs28685132^f^, rs2740351	C	miRNA Duplex Unwinding (miRISC formation)
*GSK3β*	rs3732361, rs56728675, rs60393216	C	Drosha Phosphorylation/Stabilization
*LIN28*	rs11247946, rs11587947^a^	N	Recruites ZCCHC11 to miRNA for Uridylation
*LIN28A*	rs12741800^a^, rs12728900, rs6598964, rs6697410		
*LIN28B*	rs12194974, rs17065417		Microprocessor Regulation
*MAP3K19*	rs13390171^f^, rs1551497^f^, rs2322254^f^	C	TRBP Phosphorylation/Stabilization
*MAPK1*	rs13515, rs2283792, rs6928, rs7286558, rs743409, rs8136867, rs9607272		
*MAPK14*	rs3804452, rs8510		
*MAPK3*	rs11865086		
*MAPKAPK2*	rs4240847, rs4548444, rs45514798		
*MOV10*	rs2932538	C	miRNA Duplex Unwinding (miRISC formation)
*p38 (CRK)*	rs1083	N	AGO2 Phosphorylation/P-body Localization
*p38 (GRAP)*	rs138011, rs138012		
*p54 (FKBP5)*	rs3800373, rs755658	C	Localization with P-bodies/mRNA Degradation and/or Storage
*p54 (IFIT2)*	rs17468739, rs2070845		
	rs11061209		
*PACT (PRKRA)*	rs2059691, rs1967327, rs10207436^e^	N	Associates with Dicer; Role Undetermined
*PACT (RBBP6)*	rs11860248, rs2033214, rs7195386		
*RAN*	rs10848237, rs14035, rs3809142, rs10773831	Both	Ran-GTP Associates with XPO5 for Export
*SMAD4*	rs948588	N	Drosha Processing Enhancement
*TGFB1*	rs1800468, rs1800470^f^, rs1800471, rs1800472	N	Recruitment of SMAD/Enhancement of Drosha
*TNRC6A (GW182)*	rs1030211, rs11639856, rs11642302, rs11644976, rs12917810, rs1633445, rs2303085, rs4788430, rs6497755,	C	P-bodies and mRNA Degradation
*TNRC6B*	rs7291691^a^		
*TRBP (NCOA6)*	rs4911442, rs6088619, rs910871	C	Pre-miRNA Cleavage
*TRBP (NPFF)*	rs8192593		
*TRBP (TARBP2)*	rs34649330, rs784567		
*XPO5*	rs1106841, rs11077, rs17287964, rs2257082, rs699937, rs7755135	Both	Nuclear Export of Pre-miRNA to Cytoplasm
*ZCCHC11 (TUT4)*	rs2274147, rs835036	C	Uridylation of Pre-miRNA/Dicer Inhibition

^a^ Related SNPs are those in high linkage disequilibrium (r^b^ > 0.8). These are: rs7460 (rs11996715); rs197381 (rs197383); rs13175906 (rs13186629); rs2330696 (rs6450839); rs6883386 (rs2161006, rs17404622); rs11587947 (rs11581746); rs12741800 (rs3811463); rs7291691 (rs9623117)

^b^ Some of these genes reflect actual location (SNP is within gene), others reflect literature associations

^c^ AGO genes are also known by EIF2C (i.e.AGO1 is also known by EIF2C1, etc.…)

^d^ N: Nucleus; C: Cytoplasm

^e^dbSNP lists this SNP’s position as LOC101927027; it is associated in the literature with PRKRA in the literature (falling within an accepted range of PRKRA)

^f^ These SNPs were either not evaluated for association with CRC due to unavailability of GWAS data

#### Statistical analysis

Our sample consisted of 401 cases who had both miRNA and SNP data and 1115 cases and 1173 controls with SNP data for colon cancer risk assessment. We used a logistic regression model adjusted for age, sex and study center to identify SNPs associated with colon cancer risk; we report odds ratios (OR) and 95 % confidence intervals (CI) from those models. We adjusted for multiple comparisons using the step-down Bonferroni correction [39] based upon the effective number of independent SNPs per chromosome as determined using the SNP spectral decomposition method proposed by Nyholt [40] and modified by Li and Ji [41]. SNPs that were significantly associated with colon cancer risk were then assigned either a dominant or recessive model. SNPs were also compared with mRNA RNA-Seq expression data. Out of the 219 candidate SNPs, 215 had sufficient variant allele expression to evaluate with mRNA expression. We required at least one subject with RNA-Seq expression in both tumor and normal tissue and to have the heterozygous genotype to evaluate a dominant model or the homozygous variant genotype to evaluate both the dominant and recessive models. The means of the mRNA expression data for each tissue type as measured by the log base 2 of the RPKM (Reads per Kilobase per Million) were compared in both the dominant and recessive (where possible) genotypes of each SNP. The *p*-values are based upon 10,000 permutations of the genotype using the coin package in R, and the results were adjusted for multiple comparisons at the chromosomal level using the false discovery rate (FDR) [42] level of 0.05. After adjustment for multiple comparisons, any significant SNPs were then combined with the SNPs found to be significantly associated with colorectal cancer risk and evaluated for associations with miRNA expression levels in normal colonic mucosa as well as with differential miRNA expression between carcinoma tissue and normal colonic mucosa. We compared log base 2 transformed expression levels across selected genotype models using the significance analysis of microarrays (SAM) technique in the R package siggenes [43], *p*-values were based upon 1000 permutations with an FDR level of 0.10. For siggenes, SNPs were evaluated as either dominant or recessive based on previous findings, since a two-level outcome is more interpretable. Bioinformatics analysis utilized UCSC Table Browser [44] to obtain all SNP coordinates; all coordinates are from the GRCh37 assembly.

## Results

Eleven SNPs were associated (*P* < 0.05) with colon cancer risk (Table 2). After adjustment for multiple comparisons, two of these SNPs remained significant (P_Holm_ < 0.05): rs10207436 (*PRKRA*) (OR_AG/GG_ = 0.78, 95 % CI 0.66–0.92) and rs4548444 (*MAPKAP2*) (OR_GG_ = 1.67, 95 % CI 1.12–2.48). For rs3178250 (*BMP2*) (OR_TC/CC_ = 1.18, 95 % CI 0.99–1.40) we observed that the heterozygote genotype (relative to homozygous normal) was associated with ~25 % elevated colon cancer risk, while the rare homozygous variant genotype was (non-significantly) associated with reduced risk. The dominant model did not reach statistical significance. *PRKRA* rs10207436 was associated with reduced colon cancer risk (under the dominant model), while *MAPKAPK2* rs4548444 was associated with increased risk (under the recessive model).

**Table 2:** Associations between biogenesis genes and cancer risk for those SNPs that had a raw *p*-value of <0.05

	Controls		Cases						
	N	%	N	%	OR	(95 % CI)	*P*-value^a,b^	P Holm^b^	VEP Prediction
*AGO2* (rs6985156)									
GG	912	77.7	858	77.0	1.00		0.030	0.395	
GA	250	21.3	230	20.6	0.98	(0.80, 1.20)			
AA	11	0.9	27	2.4	2.59	(1.27, 5.26)			
GG/GA vs. AA	11	0.9	27	2.4	2.6	(1.28, 5.28)			Modifier
*AGO2* (rs7843258)									
CC	810	69.1	779	69.9	1.00		0.031	0.395	
CT	342	29.2	298	26.7	0.90	(0.75, 1.08)			
TT	21	1.8	38	3.4	1.87	(1.09, 3.23)			
CC/CT vs.TT	21	1.8	38	3.4	1.93	(1.12, 3.32)			Modifier
*AGO2* (rs2176397)									
CC	567	48.3	568	50.9	1.00		0.019	0.273	
CT	517	44.1	435	39.0	0.85	(0.71, 1.01)			
TT	89	7.6	112	10.0	1.26	(0.93, 1.71)			
CC/CT vs. TT	89	7.6	112	10	1.36	(1.02, 1.83)			Modifier
*AGO2* (rs4961278)									
AA	605	51.6	578	51.8	1.00		0.032	0.395	
AG	501	42.7	444	39.8	0.92	(0.78, 1.09)			
GG	67	5.7	93	8.3	1.45	(1.04, 2.03)			
AA/AG vs. GG	67	5.7	93	8.3	1.51	(1.09, 2.09)			Modifier
*BMP2* (rs3178250)									
TT	747	63.7	663	59.5	1.00		0.014	0.040	
TC	371	31.6	415	37.2	1.24	(1.04, 1.48)			
CC	55	4.7	37	3.3	0.75	(0.49, 1.16)			
TT vs.TC/CC	426	36.3	452	40.5	1.18	(0.99, 1.40)			Modifier
*DGCR8* (rs11089328)									
AA	400	34.1	432	38.7	1.00		0.009	0.066	
AG	586	50.0	491	44.0	0.76	(0.63, 0.91)			
GG	187	15.9	192	17.2	0.94	(0.74, 1.20)			
AA vs. AG/GG	773	65.9	683	61.3	0.80	(0.68, 0.96)			Modifier
*DROSHA* (rs2287584)									
TT	743	63.3	742	66.5	1.00		0.032	0.384	
TC	394	33.6	325	29.1	0.82	(0.69, 0.99)			
CC	36	3.1	48	4.3	1.33	(0.85, 2.08)			
TT/TC vs. CC	36	3.1	48	4.3	1.42	(0.91, 2.20)			Low
*DROSHA* (rs17410035)									
GG	552	47.1	468	42.0	1.00		0.014	0.181	
GT	473	40.3	518	46.5	1.29	(1.08, 1.53)			
TT	148	12.6	129	11.6	1.01	(0.77, 1.32)			
GG vs. GT/TT	621	52.9	647	58.0	1.22	(1.03, 1.44)			Modifier^c^
*GEMIN4* (rs2740348)									
CC	782	66.7	798	71.6	1.00		0.048	0.241	
CG	352	30.0	286	25.7	0.80	(0.67, 0.96)			
GG	39	3.3	31	2.8	0.78	(0.48, 1.27)			
CC vs. CG/GG	391	33.3	317	28.4	0.80	(0.67, 0.96)			Moderate
*MAPKAPK2* (rs4548444)									
AA	713	60.8	686	61.5	1.00		0.021	0.041	
AG	417	35.5	363	32.6	0.91	(0.76, 1.08)			
GG	43	3.7	66	5.9	1.61	(1.08, 2.40)			
AA/AG vs. GG	43	3.7	66	5.9	1.67	(1.12, 2.48)			Modifier
*PRKRA* (rs10207436)									
AA	616	52.5	655	58.7	1.00		0.013	0.026	
AG	480	40.9	400	35.9	0.78	(0.66, 0.93)			
GG	77	6.6	60	5.4	0.75	(0.52, 1.07)			
AA vs. AG/GG	557	47.5	460	41.3	0.78	(0.66, 0.92)			Modifier

^a^ Adjusted for age, sex and center

^b^ These *p*-values are for the co-dominant model only

^c^ This SNP is predicted to have a ‘modifier’ effect for C5orf22 by VEP

Three SNPs in total were associated with altered mRNA expression across genotypes. Two SNPs, rs2059691 (*PRKRA*) under the dominant model and rs235768 (*BMP2*) under the recessive model, were associated with altered mRNA expression in normal colonic mucosa after multiple corrections (FDR = 0.003, 0.035 respectively) (Table 3). One SNP, rs2740349 (*GEMIN4*) was significantly associated with altered mRNA expression in carcinoma tissue, under the dominant model (FDR = 0.047).

**Table 3:** SNPs associated with mRNA expression in normal mucosa, carcinoma tissue and differential across genotypes

			Tumor		*P*-values (carcinoma)		Normal		*P*-values (normal)		*P*-values (difference)		VEP prediction
Gene	SNP	Model	*N*	Mean^c^ mRNA expression	Raw	FDR	*N*	Mean mRNA expression	Raw	FDR	Raw	FDR	
*ADAR*	rs3766925	TT	38	831.03	0.047	0.749	36	426.06					Modifier
		TA/AA	33	518.97			31	306.42					
*AGO2*	rs7460^b^	AA	19	284.79	0.027	0.316	18	113.50	0.021	0.267			Modifier
		AT/TT	52	160.38			49	70.65					
*AGO2*	rs1060832	CC/CT	70	191.79			66	83.06			0.050	0.403	Modifier
		TT	1	326.00			1	23.00					
*AGO2*	rs13276958	TT/TC	56	172.75			51	88.39			0.022	0.403	Modifier
		CC	15	271.80			16	62.31					
*AGO2*	rs2176397	CC/CT	66	174.39	0.043	0.316	60	78.37					Modifier
		TT	5	448.20			7	114.71					
*AGO2*	rs2271735	CC/CA	66	205.17	0.009	0.223	61	88.84	0.019	0.267			Modifier
		AA	5	42.00			6	14.33					
*AGO2*	rs2292775	GG/GA	70	191.79			66	83.06			0.049	0.403	Modifier
		AA	1	326.00			1	23.00					
*AGO2*	rs2292780	CC/CT	70	191.79			66	83.06			0.048	0.403	Modifier
		TT	1	326.00			1	23.00					
*AGO2*	rs2293939	GG/GA	65	204.00	0.039	0.316	60	85.65					Low
		AA	6	81.83			7	52.29					
*AGO2*	rs2944760	TT/TG	67	190.13			63	84.56			0.038	0.403	Modifier
		GG	4	253.00			4	44.50					
*AGO2*	rs2944761	GG	18	272.83	0.039	0.316	17	104.76	0.049	0.365			Modifier
		GA/AA	53	166.79			50	74.48					
*AGO2*	rs2977490	GG	21	161.81	0.034^a^	0.316	17	131.29	0.016	0.267			Modifier
		GA/AA	50	207.06			50	65.46					
*AGO2*	rs10087629	GG	22	300.91	0.005	0.223	21	115.14	0.009	0.267			Modifier
		GA/AA	49	145.53			46	67.11					
*BMP2*	rs15705	AA/AC	68	26.35	0.039	0.308	64	63.50					Modifier
		CC	3	43.33			3	70.33					
BMP2	rs235768	TT/TA	63	28.84			58	71.78	0.003	0.035	0.049	0.535	Moderate
		AA	8	13.13			9	12.44					
*DCP1B*	rs2240610	GG/GA	52	29.46			51	12.47	0.009	0.127			Modifier
		AA	19	31.79			16	29.00					
*DCP1B*	rs6489338	GG/GA	54	28.48			53	12.30	0.004	0.114			Modifier
		AA	17	35.18			14	32.00					
*DDX5*	rs1140409	AA	68	710.12	0.044	0.292	64	562.19					Moderate
		AC/CC	3	212.67			3	88.33					
*DHX16*	rs3130000	GG	60	80.68			55	63.58			0.045	0.696	Modifier
		GA/AA	11	64.36			12	74.08					
*DICER1*	rs4513027	AA/AG	65	348.18			61	286.57			0.047	0.554	Modifier
		GG	6	290.00			6	64.50					
*DROSHA*	rs573010	CC	38	230.03			37	87.57			0.038	0.769	Modifier
		CA/AA	33	172.42			30	108.30					
*DROSHA*	rs673019	AA	61	221.62	0.015	0.361	60	102.13					Modifier
		AG/GG	10	91.20			7	51.57					
*DROSHA*	rs2330696	GG	46	224.61			40	88.18			0.008	0.470	Modifier
		GA/AA	25	163.96			27	109.70					
*DROSHA*	rs3792830	AA/AG	70	200.31	0.028	0.361	66	95.74					Modifier
		GG	1	409.00			1	170.00					
*DROSHA*	rs3805525	TT/TC	61	207.46			57	87.16	0.050	0.569	0.026	0.769	Modifier
		CC	10	177.60			10	152.10					
*DROSHA*	rs6878195	TT	23	174.43			20	56.10	0.020	0.569			Modifier
		TC/CC	48	217.06			47	114.19					
*DROSHA*	rs7719666	CC/CT	49	214.47			48	112.17	0.034	0.569			Modifier
		TT	22	178.27			19	58.16					
*DROSHA*	rs10052174	AA/AG	70	200.31	0.030	0.361	66	95.74					Modifier
		GG	1	409.00			1	170.00					
*DROSHA*	rs10067066	TT/TC	70	200.31	0.028	0.361	66	95.74					Modifier
		CC	1	409.00			1	170.00					
*DROSHA*	rs13183642	GG	44	169.70			39	79.23	0.030	0.569			Modifier
		GT/TT	27	257.93			28	121.39					
*DROSHA*	rs16901165	GG/GA	70	200.31	0.029	0.361	66	95.74					Modifier
		AA	1	409.00			1	170.00					
*FAM57A*	rs2740351	AA/AG	58	42.79			54	20.30	0.006	0.089			Modifier
		GG	13	24.92			13	4.54					
*FKBP5*	rs755658	CC	59	122.02			56	179.54	0.042	0.232			Modifier
		CT/TT	12	82.50			11	69.09					
*GEMIN4*	rs7813	AA/AG	58	59.29			54	32.72	0.023	0.113			Modifier
		GG	13	28.62			13	8.69					
*GEMIN4*	rs2740348	CC	51	64.31			47	35.26	0.013	0.089			Moderate
		CG/GG	20	26.55			20	11.15					
GEMIN4	rs2740349	TT	51	64.31	0.005	0.047	47	35.26	0.012	0.089			Moderate
		TC/CC	20	26.55			20	11.15					
*MAPK1*	rs743409	GG/GA	60	280.25			56	201.55			0.032	0.979	Modifier
		AA	11	341.64			11	126.00					
*MAPK14*	rs8510	CC	60	246.67			56	147.52	0.016	0.157			Modifier
		CT/TT	11	193.64			11	62.91					
PRKRA	rs2059691^b^	GG	33	48.67	0.044^a^	0.428	30	15.37	0.000	0.003			Modifier
		GA/AA	38	89.16			37	51.24					
*RBBP6*	rs2033214	TT	58	200.36			56	130.27	0.038	0.418			Modifier
		TG/GG	13	119.23			11	51.00					
*SKIV2L*	rs449643	CC	52	73.13	0.016	0.458	50	51.50					Moderate
		CT/TT	19	136.58			17	95.35					
*TARBP2*	rs34649330	CC/CT	69	22.33	0.037	0.685	65	10.69			0.024	0.640	Modifier
		TT	2	51.50			2	10.00					
*TNRC6A*	rs1030211	AA	56	266.98	0.044	0.299	55	228.71					Modifier
		AG/GG	15	189.33			12	138.83					
*TNRC6A*	rs4788430	GG	41	298.59	0.010	0.212	39	252.33					Modifier
		GA/AA	30	184.97			28	157.29					
*TNRC6A*	rs6497755	AA	23	316.39	0.030	0.299	22	303.50	0.009	0.194			Modifier
		AC/CC	48	219.04			45	168.18					
*XPO5*	rs11077	TT/TG	57	178.12			55	70.96	0.017	0.157			Modifier
		GG	14	328.86			12	134.83					
*XPO5*	rs1106841	AA/AC	59	183.85			57	73.46	0.036	0.232			Low
		CC	12	325.83			10	133.40					
*XPO5*	rs2257082	GG/GA	66	208.95			62	75.56	0.012	0.157			Low
		AA	5	193.20			5	167.20					

^a^ This *p*-value is for the recessive model

^b^ This mRNA was significantly differentially expressed across genotypes using both the recessive and dominant models

^c^ Means are displayed in count data (rather than RPKMs)

Results from VEP showed that five SNPs within four genes had a predicted ‘moderate’ effect, meaning that the SNP is a “non-disruptive variant that might change protein effectiveness” [36]. These SNPs were: rs235768 (*BMP2*), rs2740348 and rs2740349 (*GEMIN4*) (which are in high Linkage Disequilibrium (LD) but both included for their separate results in Tables 3 and 4), rs449643 (*SKIV2L*) and rs1140409 (*DDX5*). Two SNPs, rs1106841 and rs2257082 (*XPO5*), had a predicted ‘low’ effect, meaning that they are assumed “to be mostly harmless or unlikely to change protein behavior” [36]. All other SNPs were categorized as having a ‘modifier’ impact, meaning that there is little evidence of impact due to the SNPs being in non-coding regions [36].

**Table 4:** SNPs, previously identified as significantly associated with either mRNA expression or cancer after multiple comparisons, associated with miRNA expression at an FDR level of 0.10

SNP	miRNA	*N*	Mean miRNA expression	*N*	Mean miRNA expression	*P*-values^d^
Normal Colonic Mucosa						
		AA/AG		GG		
rs4548444 (*MAPKAPK2*)^b^	hsa-miR-3163	376	9.46	25	11.35	0.0033
	hsa-miR-378c	376	7.03	25	8.72	0.0084
	hsa-miR-4316	376	6.74	25	8.22	0.0003
	hsa-miR-4444	376	8.51	25	9.85	0.0035
	hsa-miR-4753-5p	376	7.84	25	9.50	0.0059
	hsa-miR-509-5p	376	7.73	25	9.35	0.0040
	hsa-miR-5195-5p	376	8.24	25	10.16	0.0076
	hsa-miR-659-3p	376	10.83	25	12.85	0.0095
	hsa-miR-770-5p	376	5.93	25	7.11	0.0030
		TT		TC/CC		
rs2740349 (*GEMIN4*)^a^	hsa-miR-145-5p	280	184.32	121	229.78	0.0011
	hsa-miR-378a-3p	280	145.01	121	163.22	0.0005
	hsa-miR-378i	280	72.24	121	80.89	0.0013
	hsa-miR-4428	280	1322.42	121	1442.51	0.0017
	hsa-miR-451a	280	23.52	121	41.62	0.0021
	hsa-miR-494	280	15338.94	121	16994.17	0.0013
		TT/TA		AA		
rs235768 (*BMP2*)^a^	hsa-miR-1321	357	4.89	44	6.17	0.0034
	hsa-miR-206	357	10.43	44	12.25	0.0032
	hsa-miR-23a-5p	357	5.98	44	6.61	0.0045
	hsa-miR-30c-2-3p	357	4.70	44	5.51	0.0001
	hsa-miR-3189-3p	357	6.49	44	7.27	0.0032
	hsa-miR-4494	357	9.63	44	10.62	0.0021
	hsa-miR-4648	357	9.00	44	9.99	0.0017
	hsa-miR-4707-3p	357	8.05	44	9.75	0.0001
Differential (between carcinoma tissue and normal colonic mucosa)						
		AA		AG/GG		
rs11089328 (*DGCR8*)^c^	hsa-miR-645	140	1.27	243	2.14	0.0002

^a^ SNP significantly associated with mRNA expression after correction for multiple comparisons

^b^ SNP significantly associated with cancer after correction for multiple comparisons

^c^ SNP associated with cancer, but not significantly after correction for multiple comparisons

^d^ Raw *p*-value

Twenty-four unique miRNAs were dysregulated in total with SNPs that were either significantly associated with altered mRNA expression or cancer risk. Twenty-three of these were seen in normal colonic mucosa across genotypes; eight of these were associated with rs4548444 (*MAPKAPK2*) under the recessive genotype, six with rs2740349 (*GEMIN4*) under the dominant genotype, and nine with rs235768 (*BMP2*) under the recessive genotype (Table 4). One miRNA, hsa-miR-645, was seen to be significantly associated with rs11089328 (*DGCR8*) in differential tissue expression under the dominant genotype.

## Discussion

As the vast majority of mature miRNAs are generated by a group of genes either directly or indirectly related to miRNA biogenesis, SNPs within these genes could alter miRNA expression and subsequently colon cancer risk. Of the 219 SNPs within the miRNA biogenesis-related genes we analyzed, 11 SNPs were significantly associated with colon cancer. Three of these were significantly associated with colon cancer after correction for multiple comparisons. Of these, only *DGCR8* rs11089328 and *MAPKAPK2* rs4548444, were associated with altered miRNA expression when an FDR level of 0.1 was applied.

We previously reported, in a study with a larger sample size, that rs3178250 (*BMP2*) was associated with increased risk (OR_TC/CC_ 1.20 95 % CI 1.05, 1.38) of developing colon cancer, as well as rectal cancer (OR_CC_ 1.63 95 % CI 1.02, 2.60) [45]. The larger sample of approximately 500 cases provided adequate power to detect a significant association for this SNP. BMP2 is a member of the TGF-β superfamily which has been associated with colorectal cancer [46]. Currently to our knowledge there are no significant associations between the other significant SNPs we identified and colorectal cancer in the literature.

Of the 212 SNPs evaluated with mRNA expression, 48 SNPs within 18 genes were associated with altered mRNA expression in either normal colonic mucosa, carcinoma tissue or difference in expression between normal colonic mucosa and carcinoma tissue; three of these remained significant after adjustment for multiple comparisons. One of these three SNPs, rs2740349 (*GEMIN4*), was associated with altered mRNA expression in carcinoma tissue, and the two other SNPs, rs2059691 (*PRKRA*) and rs235768 (*BMP2*), were associated with altered levels of mRNA expression in normal colonic mucosa across genotypes. Both rs2740349 (*GEMIN4*), under the dominant model and rs235768 (*BMP2*), under the recessive model, had less mRNA expression with the variant allele. Rs2059691 (*PRKRA*) had more expression in the GA/AA genotype as compared to the GG rarer genotype. While rs2740349 (*GEMIN4*) and rs235768 (*BMP2*) were both predicted by VEP to have a ‘moderate’ effect on the host gene, rs2059691 (*PRKRA*) was predicted to have a ‘modifier’ effect. Subsequently, rs2740349 (*GEMIN4*) and rs235768 (*BMP2*) were both shown to be associated with altered levels of miRNA expression and in both cases all miRNAs were upregulated in the corresponding variant genotypes. Conversely, rs2059691 (*PRKRA*) was not predicted to have any effect, and we saw increased levels of mRNA expression in the variant genotype, and no subsequent miRNA expression associations.

*MAPKAPK2* rs4548444 was associated with an increase in mean miRNA expression in normal colonic mucosa tissue, however this increase was seen with only the homozygote rare genotype. This SNP is associated with increased risk of colon cancer (OR_GG_ 1.61 95 % CI 1.08, 2.40). MAPK/ERK proteins have been shown to phosphorylate TRBP, stabilizing it and potentially increasing Dicer-mediated processing of pre-miRNAs. A SNP in *MAPKAPK2* could cause an altered binding affinity of MAPKAPK2 to TRBP, thereby impacting mature miRNA production. As we did not see any association between rs4548444 and *MAPKAPK2* mRNA expression, and we saw an increase in miRNA expression in the GG (homozygous rare) genotype, this indicates that while the SNP is associated with colon cancer risk and miRNA expression, it is not doing so through altered miRNA biogenesis. *MAPKAPK2* is involved in many cellular processes, including stress and inflammatory response, gene regulation and cell proliferation, and nuclear export [47]; as such, it is possible that the increased risk of colon cancer is associated with another biological process that *MAPKAPK2* regulates.

Using Ensembl’s VEP to predict the effect of each SNP on its corresponding gene showed that very few SNPs were predicted to have any effect. There were five SNPs within four genes that had a ‘moderate’ effect. Of these, only one was seen to be associated with altered colon cancer risk, rs2740348 (*GEMIN4*), which is in LD with rs2740349 (*GEMIN4*). While there were no significant associations were seen between rs2740348 and miRNA expression or mRNA expression, we did see significant findings after correction for multiple comparisons for mRNA and for miRNA expression with rs2740349 (*GEMIN4*). However, miRNA expression was upregulated in the same genotype with which mRNA expression was downregulated; this suggests that this SNP increases miRNA expression. Because the helicase GEMIN4 is thought to aid in miRNA duplex unwinding [12], a reduction in *GEMIN4* transcription should theoretically result in reduced miRNA biogenesis. Similarly, BMPs have been thought to enhance Drosha cleavage [12], thereby increasing mature miRNA levels. As we see that the opposite is the case, and that miRNA levels increase with the reduction of *BMP2* and *GEMIN4* transcription, it is possible that other mechanisms influence miRNA biogenesis, or these genes have additional roles in biogenesis.

Interestingly, all of the miRNAs that were dysregulated were unique, in that no one miRNA was associated with more than one SNP. None of these miRNAs is listed as a known target in miRTarBase, a repository of validated miRNA target associations (http://mirtarbase.mbc.nctu.edu.tw/) [48] at this time, and there are no commonalities of chromosomal location between the SNPs and their respectively associated miRNAs. This suggests that miRNA biogenesis may be specific to the level of the individual miRNA, and the reason these specific groups of miRNAs are dysregulated across genotypes is because these genes affect these specific miRNA’s production. Winter et al. describe findings to support that “specific helicases may regulate miRNAs differentially” [12]; this would support subsets of miRNAs associated with different SNPs. Perhaps other proteins have a similar influence on specific miRNA biogenesis.

Biogenesis genes contribute to the processing of virtually every mature miRNA, however miRNA expression is thought to be tissue specific. One limitation of our study is that our findings could be influenced by our use of tissue from colon cancer patients, evaluating both normal colonic mucosa as well as differentially expressed miRNAs between carcinoma and normal colonic mucosa. Since we only evaluated associations with miRNA expression levels for SNPs associated with colon cancer risk or mRNA expression, other SNPs in biogenesis genes could alter miRNAs in other situations. It should also be kept in mind that many of the accessory genes to miRNA biogenesis are involved in multiple biological pathways. Thus, it is possible that SNPs could alter colon cancer risk through multiple mechanisms. Additionally, because we only looked at more commonly occurring SNPs (MAF ≥0.01) and we required there to be at least one subject in our dataset with the homogeneous variant genotype in order to analyze the dominant and recessive genotypes and at least one subject with the heterozygous genotype to evaluate the dominant model and overall CRC risk, another possible limitation is that more rare, and possibly more deleterious, SNP associations were not evaluated in this study.

We hypothesized that SNPs within biogenesis genes could impact the transcription of these mRNAs, and therefore impact miRNA expression. Out of the 212 SNPs evaluated with mRNA expression only three were significantly associated with altered expression after correction for multiple comparisons. This indicates that, in colon cancer, the majority of these SNPs do not negatively impact biogenesis gene transcription. While other mechanisms could still alter cellular protein levels, and thus possibly miRNA biogenesis, within the cell, we are not able to measure this. We also hypothesized that SNPs in biogenesis genes would be associated colon cancer risk and that miRNA expression levels would be influenced by the genotype of these SNPs. However, out of 219 SNPs within 48 genes, only one SNP was associated with both altered colon cancer risk and altered miRNA expression levels. Because the SNP that was associated with both colon cancer risk and miRNA expression belongs to the MAPK family, and as such is involved in many processes other than miRNA biogenesis, the impact on cancer and even on miRNA expression may be due to other mechanisms unrelated to miRNA biogenesis. This finding suggests that SNPs in miRNA biogenesis genes have minimal impact on colon cancer risk and those that were associated have minimal associations with miRNAs.

## Conclusion

Our data suggest that few of the SNPs in biogenesis genes we evaluated alter levels of mRNA transcription or colon cancer risk. As only one SNP both alters colon cancer risk and miRNA expression it is likely that SNPs influencing cancer do not do so through miRNAs. Because the significant SNPs were associated with downregulated mRNAs and upregulated miRNAs, and because each SNP was associated with unique miRNAs, it is possible that other mechanisms influence mature miRNA levels.

### Ethics and consent to participate

All participants signed an informed consent and this study was approved by the Institutional Review Board at the University of Utah; the committee numbers for this paper are IRB_00055877 and IRB_00002335.

### Consent to publish

Not applicable.

### Availability of data and materials

Utah SNP data are available in NCBI’s dbGaP repository (http://www.ncbi.nlm.nih.gov/gap) under the accession number phs000410.v1.p1. Due to restrictions in the signed consent forms, microarray data cannot be released at this time.

## Abbreviations

ADAR: Adenosine-Deaminase, RNA-specific
AGO: Argonaute
BMP: bone morphogenetic protein
bp: base pair
cDNA: complementary DNA
CI: confidence intervals
CRC: colorectal cancer
DGCR8: DiGeorge Syndrome Critical Region 8
ERK: extracellular regulated kinase
FDR: false discovery rate
FMRP: fragile X mental retardation protein
GWAS: Genome Wide Association Study
kb: kilobase
KPMRP: Kaiser Permanente Medical Research Program
LD: Linkage disequilibrium
MAF: minor allele frequency
MAPK: mitogen-activated protein kinase
miRNA: microRNA
mRNA: messenger RNA
ng: nanogram
nt: nucleotide
OR: odds ratios
pre-miRNA: precursor miRNA
pri-miRNA: primary-microRNA
QC: quality control
qRT-PCR: quantitative reverse transcription polymerase chain reaction
RISC: RNA-induced silencing complex
RNase: ribonuclease
RPKM: Reads per Kilobase per Million
SAM: significance analysis of microarray
SNP: single nucleotide polymorphism
TGF-β: transforming growth factor-β
TRBP: trans-activating response RNA binding protein
TRNC: trinucleotide repeat-containing protein
TUT4: terminal uridine transferase
VEP: variant effect predictor
XPO5: exportin-5
